# Occurrence and molecular epidemiology of *Giardia duodenalis* infection in dog populations in eastern Spain

**DOI:** 10.1186/s12917-018-1353-z

**Published:** 2018-01-22

**Authors:** Manuel Adell-Aledón, Pamela C. Köster, Aida de Lucio, Paula Puente, Marta Hernández-de-Mingo, Paula Sánchez-Thevenet, María Auxiliadora Dea-Ayuela, David Carmena

**Affiliations:** 1 CEU Cardenal Herrera University, C/ Grecia, 31, 12006 Castellón de la Plana, Castellón Spain; 2 Parasitology Reference and Research Laboratory, National Centre for Microbiology, Health Institute Carlos III, Ctra. Majadahonda-Pozuelo Km 2, 28220 Majadahonda, Madrid Spain; 3 CEU Cardenal Herrera University, C/ Luis Vives 1, 46115 Alfara del Patriarca, Valencia Spain

**Keywords:** *Giardia duodenalis*, Protozoa, Dogs, Molecular epidemiology, Castellón, Spain

## Abstract

**Background:**

*Giardia duodenalis* is one of the most common enteric parasites in domestic animals including dogs. Young animals are more prone to the infection, with clinical manifestations ranging from asymptomatic to acute or chronic diarrhoea. Dogs are primarily infected by canine-specific (C-D) assemblages of *G. duodenalis*. However, zoonotic assemblages A and B have been increasingly documented in canine isolates, raising the question of whether and to which extent dogs can act as natural reservoirs of human giardiosis.

**Methods:**

In this cross-sectional epidemiological survey we assessed the molecular diversity of *G. duodenalis* in dogs in the province of Castellón, Eastern Spain. A total of 348 individual faecal samples from sheltered (*n* = 218), breeding (*n* = 24), hunting (*n* = 68), shepherd (*n* = 24), and pet (*n* = 14) dogs were collected between 2014 and 2016. Detection of *G. duodenalis* cysts in faecal material was carried out by direct fluorescence microscopy as a screening test, whereas a qPCR targeting the small subunit ribosomal RNA gene of the parasite was subsequently used as a confirmatory method.

**Results:**

*Giardia duodenalis* was detected in 36.5% (95% CI: 31.6–41.7%) of dogs. No significant differences in prevalence rates could be demonstrated among dogs according to their sex and geographical origin, but breeding (45.8%; 95% CI: 27.9–64.9%) and sheltered (40.4%; 95% CI: 34.1–47.0%) dogs harboured significantly higher proportions of *G. duodenalis*. Multi-locus sequence-based genotyping of the glutamate dehydrogenase and β-giardin genes of *G. duodenalis* allowed the characterization of 35 canine isolates that were unambiguously assigned to assemblages A (14.3%), B (22.9%), C (5.7%), and D (37.1%). A number of inter-assemblage mixed infections including A + B (11.4%), A + D (2.9%), and A + B + D (5.7%) were also identified.

**Conclusions:**

Data presented here are strongly indicative of high infection pressures in kennelled animals. Zoonotic sub-assemblages AII, BIII, and BIV were responsible for a considerable proportion of the *G. duodenalis* infections detected, but very few of the genotypes identified have been previously documented in Spanish human populations. Although possible, zoonotic transmission between dogs and humans seems an infrequent event in this Spanish region.

**Electronic supplementary material:**

The online version of this article (10.1186/s12917-018-1353-z) contains supplementary material, which is available to authorized users.

## Background

Pet animals in general and dogs in particular are increasingly regarded as true family members in many homes globally. In the UK in 2016 canine population stood at around 8.5 million, with 24% households owning a dog [1]. Since the early 1980s, the human-canine bonding has been demonstrated to significantly improve the health and well-being of both people and dogs by strengthening emotional, psychological, and physical interactions [2]. Indeed, dogs are nowadays successfully used in hospital-based animal assisted therapy programs [3]. However, dogs can act as natural reservoirs of a number of zoonotic parasitic infections including leishmaniosis, giardiosis, cryptosporidiosis, echinococcosis, dirofilariosis, and toxocariosis, particularly if improperly cared for or mistreated [4, 5].

The enteric protozoan parasite *Giardia duodenalis* is one of the most commonly detected pathogens associated with diarrhoea in humans and animals, including domestic dogs [6, 7]. As in other host species, canine infections by *G. duodenalis* can present with a broad range of clinical manifestations from asymptomatic to acute or chronic disease [8, 9]. *Giardia duodenalis* is currently regarded as a complex of eight (A-H) genotypes, also known as assemblages, displaying distinct host specificities and transmission patterns. Assemblages A and B have the widest host ranges, infecting humans, domestic animals and livestock and a large number of wildlife species, and are therefore considered zoonotic. On the contrary, assemblages C-H appear to infect a far more limited number of host species. Thus, assemblages C and D are found mainly in dogs, assemblage E in hoofed animals, assemblage F in cats, assemblage G in rodents, and assemblage H in marine mammals [10, 11].

The potential role of domestic dogs as a source of human giardiosis has been a topic of intense debate and research in the last years, with still uncertain conclusions [6]. Whereas large household- or community based surveys conducted in Cambodia [12], Peru [13], and Spain [14] concluded that dogs play a minor or no role at all in the transmission of *G. duodenalis* infections to humans, other studies suggested that transmission would be possible under favourable epidemiological conditions [15, 16].

In Spain, the presence of *G. duodenalis* in canine populations has been investigated in a limited number of epidemiological surveys using coprological examination by either conventional or direct immunofluorescence microscopy, post-mortem examination, or PCR-based methods [7, 17]. Giardiosis has been documented at infection rates of 6–38% in sheltered and hunting dogs in Barcelona [18, 19], of 1–33% in sheltered dogs in Córdoba and Álava [20, 21], and of 7–16% in sheltered and stray dogs in Murcia and Madrid [22–24]. *Giardia duodenalis* cysts have also been identified in soil samples from public parks in Madrid [25], but not in Córdoba [20]. Regarding the potential zoonotic transmission of the parasite, dog ownership has been linked with an increase in the prevalence odds of human giardiosis in Álava [26], although no dog-human transmission could be demonstrated in a recent household-based survey in the same geographical area [14]. Genotyping data are even scarcer, being only reported in few molecular studies carried out in Madrid [24], Catalonia [19], and the Basque Country [14, 21].

In this molecular epidemiological survey we present novel data on the presence, molecular diversity and frequency of *G. duodenalis* in different dog populations in Castellón, a geographical region where the occurrence of this protozoan parasite had not been previously studied. Molecular data on the *G. duodenalis* assemblages and sub-assemblages found was used to evaluate the potential role of domestic dogs as suitable reservoirs of human giardiosis.

## Methods

### Study area

**Fig. 1:**
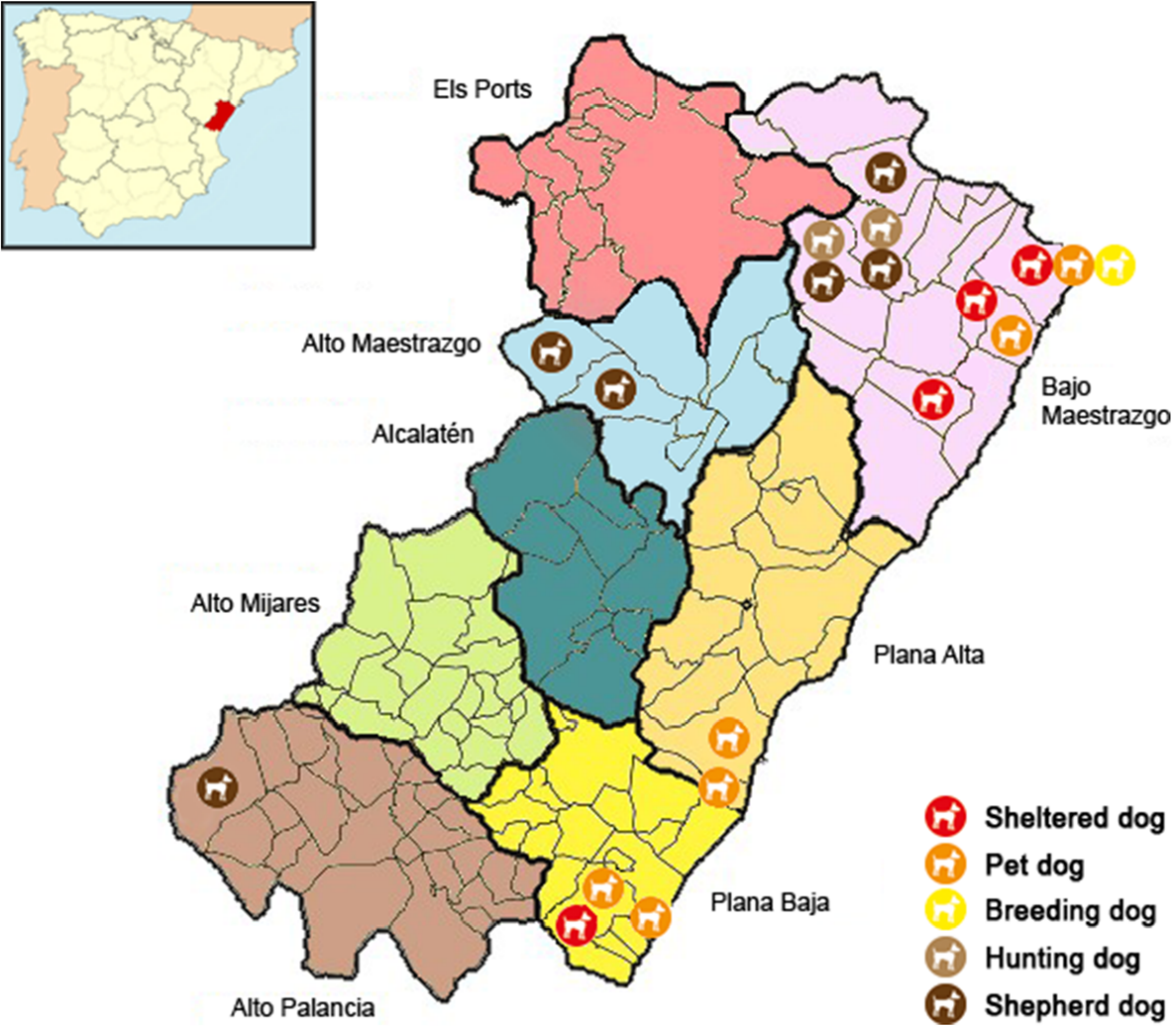
Map of the administrative divisions of the province of Castellón. The municipalities where sampling was conducted and the status of the dog sub-populations are indicated. The location of Castellón in Spain is highlighted in red in the upper left corner. Images used are in the public domain and have been downloaded from https://eswikipediaorg/wiki/Anexo:Municipios_de_la_provincia_de_Castell%C3%B3n

Castellón, one of the three provinces forming the Autonomous Region of Valencia in Eastern Spain, extends over 6632 km^2^ and has a total population of 582,327 inhabitants, a third of them living in the capital city Castellón de la Plana [27]. The province is divided in 135 municipalities distributed in eight administrative regions (Fig. 1). There are officially 151,311 domestic dogs in Castellón [28]. Of them, a total of 21,936 dogs belong to hound-type breeds commonly used in hunting, whereas an undetermined number of guard or shepherd dogs are used in agricultural exploitations in rural areas [28]. Out of the five (all privately owned) animal shelters operating in Castellón, four agreed to participate in this survey. One of them was subcontracted by the town hall, managing most stray, abandoned, or surrendered animals in the province. Besides providing attention and care, this centre was also a licensed breeding kennel and developed adoption programs to find new homes for the collected dogs. Owners of hunting, shepherd, and pet dogs were personally contacted and requested to voluntary participate in the present survey.

### Faecal sample collection

A total of 348 faecal dropping samples from individual dogs were collected during the period July 2014 and July 2016. Faecal specimens belonging to dogs from animal shelters (*n* = 218) or breeding dogs which were for sale (*n* = 24) were collected at the time of daily box cleaning. Faecal samples from hunting (*n* = 68), shepherd (*n* = 24), and pet (*n* = 14) dogs were collected just or soon after defecation. In all cases faecal samples were obtained within 24 h of excretion. Obtained faecal specimens were placed in screw-topped specimen containers and uniquely labelled indicating identification number and date of collection. Information regarding sex, status, and geographical origin of the animal was also recorded. Age data was only available for a limited number of animals and therefore this variable was not included in our analyses. Faecal specimens were transported in refrigerated boxes to the laboratory at the CEU Cardenal Herrera University (Castellón), stored at 4 °C, and processed within 24 h of collection. Two aliquots of each faecal sample were prepared as follows: i) one was re-suspended in 5–10 mL of 10% formal saline solution and kept until examination by fluorescence microscopy; ii) the other was preserved in 70% ethanol and shipped to the Parasitology Reference and Research Laboratory, Spanish National Centre for Microbiology (Majadahonda) for further molecular analyses.

### Direct fluorescent antibody test

A direct fluorescent antibody test (DFAT) was used to detect *Giardia* cysts by fluorescence microscopy. Briefly, faecal material was processed using the formalin-ethyl acetate sedimentation concentration method as described elsewhere [22]. Ten μL of concentrated faecal material were placed on welled slides. Smears were air-dried, methanol fixed, stained with fluorescein-labelled mouse monoclonal antibodies (Merifluor® Cryptosporidium/Giardia, Meridian Bioscience, OH, USA), and examined at 400× magnification.

### DNA extraction and purification

Total DNA was extracted from those faecal samples that tested positive by DFAT. An aliquot (~ 200 mg) of each faecal sample in 70% ethanol was processed using the QIAamp® DNA Stool Mini Kit (QIAGEN, Hilden, Germany) according to the manufacturer’s instructions. Purified DNA samples (200 μL) were stored at − 20 °C for further downstream molecular analysis. A water extraction control was routinely included in each sample batch processed.

### Molecular detection of *Giardia duodenalis*

Detection of *G. duodenalis* DNA was achieved using a real-time PCR (qPCR) method targeting a 62-bp region of the small subunit ribosomal RNA (*ssu* rRNA) gene of the parasite [29]. Amplification reactions were conducted in a volume of 25 μL containing 3 μL of template DNA, 12.5 pmol of primers Gd-80F and Gd-127R, 10 pmol of probe (Additional file 1: Table S1), and 12.5 μL TaqMan® Gene Expression Master Mix (Applied Biosystems, CA, USA). Detection of parasitic DNA was performed on a Corbett Rotor-Gene 6000 real-time PCR cycler (Qiagen Corbett, Hilden, Germany) using an amplification protocol consisting of an initial hold step of 2 min at 55 °C and 15 min at 95 °C followed by 45 cycles of 15 s at 95 °C and 1 min at 60 °C. The ramping of the machine was 10 °C/s in every step. No-template water (negative) and DNA (positive) controls of genomic DNA were included in each PCR run.

### Molecular characterization of *Giardia duodenalis* isolates

*Giardia duodenalis* isolates that tested positive by real-time PCR were subsequently assessed at the glutamate dehydrogenase (*gdh*) and ß-giardin (*bg*) loci. A semi-nested-PCR protocol was used to amplify a ~ 432-bp fragment of the *gdh* gene [30]. PCR reaction mixtures (25 μL) consisted of 5 μL of template DNA, 0.5 μM of each primer (GDHeF/GDHiR in the primary reaction and GDHiF/GDHiR in the secondary reaction, respectively, Additional file 1: Table S1), 2.5 units of MyTAQ™ DNA polymerase (Bioline GmbH, Luckenwalde, Germany), and 5 μL of MyTAQ™ Reaction Buffer containing 5 mM dNTPs and 15 mM MgCl_2_. Both amplification protocols consisted of an initial denaturation step at 95 °C for 3 min, followed by 35 cycles of 95 °C for 30 s, 55 °C for 30 s and 72 °C for 1 min, with a final extension of 72 °C.

Similarly, a ~ 511-bp fragment of the *bg* gene of *G. duodenalis* was amplified using a nested-PCR protocol [31]. PCR reaction mixtures (25 μL) consisted of 3 μL of template DNA, 0.4 μM of each primer (G7_F/G759_R in the primary reaction and G99_F/ G609_R in the secondary reaction, respectively, Additional file 1: Table S1), 2.5 units of MyTAQ™ DNA polymerase (Bioline GmbH), and 5 μL of MyTAQ™ Reaction Buffer containing 5 mM dNTPs and 15 mM MgCl_2_. The primary PCR reaction was carried out with the following amplification conditions: 1 cycle of 95 °C for 7 min, followed by 35 cycles of 95 °C for 30 s, 65 °C for 30 s, and 72 °C for 1 min with a final extension of 72 °C for 7 min. The conditions for the secondary PCR were identical to the primary PCR except that the annealing temperature was 55 °C.

PCR reactions were carried out on a 2720 thermal cycler (Applied Biosystems). Laboratory-confirmed positive and negative DNA samples were routinely used as controls and included in each round of PCR. PCR amplicons were visualized on 2% D5 agarose gels (Conda, Madrid, Spain) stained with Pronasafe nucleic acid staining solution (Conda). Positive-PCR products were directly sequenced in both directions using the internal primer set described above. DNA sequencing was conducted by capillary electrophoresis using the BigDye® Terminator chemistry (Applied Biosystems).

### Data analyses

The chi-square test was used to compare parasite prevalence rates in the canine population under study by sex, status (sheltered, breeding, pet, hunting or shepherd) and geographical origin of the dogs. A probability (*P*) value < 0.05 was considered evidence of statistical significance. Data were analysed with the free software RStudio Version 1.0.44 (https://www.rstudio.com/) using the Epitools library.

Raw sequencing data in both forward and reverse directions were viewed using the Chromas Lite version 2.1 sequence analysis program (https://technelysium.com.au/wp/chromas/). The BLAST tool (http://blast.ncbi.nlm.nih.gov/Blast.cgi) was used to compare nucleotide sequences with sequences retrieved from the National Center for Biotechnology Information (NCBI) database. Generated DNA consensus sequences were aligned to appropriate reference sequences using the MEGA 6 free software (http://www.megasoftware.net/) to identify *Giardia* species and assemblages/sub-assemblages [32].

For the identification of the phylogenetic inferences among the identified positive samples, a phylogenetic tree was inferred using the Neighbor-Joining method in MEGA 6. The evolutionary distances were computed using the Kimura 2-parameter method, and modelled with a gamma distribution. The reliability of the phylogenetic analyses at each branch node was estimated by the bootstrap method using 1000 replications. Representative reference sequences of the different *G. duodenalis* sub-assemblages taken from the NCBI database were also included in the phylogenetic analysis for comparative purposes.

The sequences obtained in this study have been deposited in GenBank under accession numbers MF285561 to MF285603.

## Results

### Detection of *G. duodenalis* in canine faecal samples

The overall prevalence of *G. duodenalis* in the investigated dog population was estimated at 36.5% [95% Confident Interval (CI): 31.6–41.7%]. Out of the 127 dogs that tested positive by DFAT, 81.1% (103/127) were confirmed by qPCR, whereas 18.9 (24/127) failed to be detected by the latter method. The qPCR-positive samples had cycle threshold (Ct) values ranging from 21.4 to 39.6 (mean: 29.4; SD: 3.8). Overall, 68.9% (71/103) of the *Giardia*-positive isolates by qPCR had Ct values ≥30 (Additional file 2: Figure S2). Regarding consistency, all the faecal samples processed and analysed were hard and formed or soft but formed. No loose or liquid faecal samples were noticed in the studied canine population.

Table 1 shows the occurrence of *G. duodenalis* according to the sex, status, and region of origin of the surveyed dogs. The male/female ratio was 1.3. *Giardia duodenalis* was more prevalent in female dogs than in male dogs, although this difference was not statistically significant (*P* = 0.513). Regarding dog status, detected *G. duodenalis* prevalences ranged from 20.6% (95% CI, 12.7–31.6%) in hunting dogs to 45.8% (95% CI: 27.9–64.9%) in breeding dogs. Statistically significant differences were found between hunting and breeding dogs (*P* = 0.017) and between hunting and sheltered dogs (*P* = 0.003). No obvious differences in the geographical distribution pattern of *G. duodenalis* was demonstrated (*P* = 0.152), although the infection was more frequently detected in Alcalatén (80.0%; 95% CI: 37.6–96.4%) and Bajo Maestrazgo (41.7%; 95% CI: 34.7–49.1%), with Alto Maestrazgo (16.7%; 95% CI: 4.7–44.8%) and Plana Alta (29.7%; 95% CI: 21.7–39.2%) harbouring the lowest infection rates.

**Table 1:** Prevalence and 95% confidence intervals (CIs) of *Giardia duodenalis* in dogs, as determined by direct fluorescent antibody assay. Results have been categorized according to sex, status, and geographical region of origin of the investigated dogs (*n* = 348) from Castellón, Spain, 2014–2016. Chi-square determined *P*-values are indicated

Variable	No.	*Giardia duodenalis*	Percent	95% CI	*P*-value
Sex^a^					
Male	158	46	29.1	22.6–36.6	.513
Female	119	39	32.8	25.0–41.6	
Status					
Sheltered dog	218	88	40.4	34.1–47.0	.045
Breeding dog	24	11	45.8	27.9–64.9	
Pet dog	14	5	35.7	16.3–61.2	
Hunting dog	68	14	20.6	12.7–31.6	
Shepherd dog	24	9	37.5	21.2–57.3	
Origin^b^					
Alcalatén	5	4	80.0	37.6–96.4	.152^c^
Alto Palancia	10	4	40.0	16.8–68.7	
Alto Maestrazgo	12	2	16.7	4.7–44.8	
Bajo Maestrazgo	175	73	41.7	34.7–49.1	
Plana Baja	45	14	31.1	19.5–45.7	
Plana Alta	101	30	29.7	21.7–39.2	

^a^No data available from 71 dogs

^b^Place of living of the dog at the moment of sampling. For sheltered dogs the term refers to the municipality where the animal was captured or surrendered

^c^Dogs from the municipality of Alcalatén were not included in the statistical analysis because of low sample size

### Molecular characterization of *G. duodenalis* isolates

Out of the 103 *G. duodenalis* isolates confirmed by qPCR, 34.0% (35/103) were successfully amplified at the *gdh* and/or *bg* markers. Multi-locus genotyping data were produced for 28.6% (10/35) of them, whereas 51.4% (18/35) and 20.0% (7/35) of the canine isolates were only amplified at the *gdh* or the *bg* loci, respectively. Sequence analyses revealed the presence of assemblages A (14.3%; 5/35), B (22.9%; 8/35), C (5.7%; 2/35), and D (37.1%¸13/35) (Tables 2 and 3). A number of mixed infections with more than one assemblage of *G. duodenalis* interpreted as A + B (11.4%; 4/35), A + D (2.9%; 1/35), and A + B + D (5.7%; 2/35) were also identified (Table 4). Sheltered dogs harboured the widest range of *G. duodenalis* assemblages, including A, B, C, and D. Breeding and hunting dogs were found infected by assemblages A, B, and D, whereas A and B were the only assemblages identified in shepherd dogs. None of the *G. duodenalis* isolates obtained from pet dogs could be characterized at the assemblage level. Interestingly, all seven inter-assemblage mixed infections were detected in kennelled animals, three of them in breeding dogs and the remaining four in sheltered dogs (Tables 2, 3 and 4).

**Table 2:** Diversity, frequency, and molecular features of canine-derived *Giardia duodenalis* isolates at the glutamate dehydrogenase locus. Castellón, Eastern Spain, 2014–2016. GenBank accession numbers are provided. Novel genotypes are shown underlined

Assemblage	Sub-assemblage	No. isolates	Dog status	Reference sequence	Stretch (pb)	Single nucleotide polymorphism(s)	GenBank accession number
A	AII	1	Hunting	L40510	78–482	A175R, A271R	MF285561
		1	Sheltered	L40510	78–482	C198T	MF285562
B	BIII	1	Shepherd	AF069059	54–455	C87T, G93R, T95Y, C99Y, T147Y, G150R, T230Y, G277R, C309T	MF285563
		1	Sheltered	AF069059	54–455	C87T, T138Y, T147Y, T219Y, T237Y, C309Y, C330Y, G354A, G372R, T382K, G406R, A414R, G444R	MF285564
		1	Sheltered	AF069059	44–455	C87Y, C99Y, T147Y, G189R, C309T, G354R, G406R	MF285565
	BIV	1	Shepherd	L40508	80–481	T183C, T387C, C396T, C423T	MF285566
		1	Sheltered	L40508	109–496	C133A, T464Y	MF285567
C	–	1	Sheltered	U60984	78–490	No	MF285568
D	–	1	Sheltered	U60986	80–481	No	MF285569
	–	1	Sheltered	U60986	80–481	G225R, T429C, G441A,	MF285570
	–	2	Breeding, Sheltered	U60986	80–481	T240C	MF285571
	–	1	Hunting	U60986	80–481	T240C, C375T	MF285572
	–	1	Sheltered	U60986	80–481	T240Y, T429Y, G441R	MF285573
	–	3	Sheltered	U60986	80–481	T240C, T429C, G441A, T459A	MF285574
	–	1	Sheltered	U60986	80–481	C375T	MF285575
	–	2	Hunting, Sheltered	U60986	80–481	T429C, G441A	MF285576
	–	1	Sheltered	U60986	80–481	C216Y, T429C, G441A, C471Y	MF285577

K: A/T; R: A/G; Y: C/T

**Table 3:** Diversity, frequency, and molecular features of canine-derived *Giardia duodenalis* isolates at the beta-giardin locus. Castellón, Eastern Spain, 2014–2016. GenBank accession numbers are provided. Novel genotypes are shown underlined

Assemblage	Sub-assemblage	No. isolates	Dog status	Reference sequence	Stretch (pb)	Single nucleotide polymorphism(s)	GenBank accession number
A	AII	1	Hunting	AY072723	97–590	T187Y	MF285578
		1	Hunting	AY072723	102–590	A227R, G434A	MF285579
		1	Hunting	AY072723	98–590	G261A, G277A, T329A, T564C	MF285580
		1	Sheltered	AY072723	106–587	T390Y	MF285581
	AIII	1	Shepherd	AY072724	103–590	A125R, C414Y, T558Y	MF285582
B	–	1	Breeding	AY072727	104–590	G159A, C165T, C309T, C324T, C393T, T471C	MF285583
C	–	1	Sheltered	AY545646	11–500	G37A, C451T	MF285584
D	–	1	Sheltered	AY545647	105–590	No	MF285585
	–	1	Sheltered	AY545647	105–590	G129A, A201G, C207A, A455R	MF285586
	–	1	Sheltered	AY545647	105–590	A201G, C207Y	MF285587

R: A/G; Y: C/T

**Table 4:** Mixed infections and discordant typing results detected in canine-derived *Giardia duodenalis* isolates at the glutamate dehydrogenase (*gdh*) and/or the beta-giardin (*bg*) loci, Castellón, Eastern Spain, 2014–2016. GenBank accession numbers are provided

Dog status	*gdh* locus	GenBank accession number	*bg* locus	GenBank accession number	Assigned genotype
Breeding	–	–	AII + B	MF285596	AII + B
Breeding	BIV + D	MF285588	AIII+B	MF285597	AIII+BIV + D
Breeding	D	MF285589	A^a^ + B + D	MF285598	A + B + D
Sheltered	BIII/BIV	MF285590	B	MF285599	BIII/BIV
Hunting	BIII/BIV	MF285591	–	–	BIII/BIV
Hunting	BIII/BIV	MF285592	AII	MF285600	AII + BIII/BIV
Hunting	D	MF285593	AII	MF285601	AII + D
Hunting	BIV	MF285594	AII	MF285602	AII + BIV
Hunting	BIII/BIV	MF285595	AII	MF285603	AII + BIII/BIV

^a^No molecular typing at the sub-assemblage level was possible

**Fig. 2:**
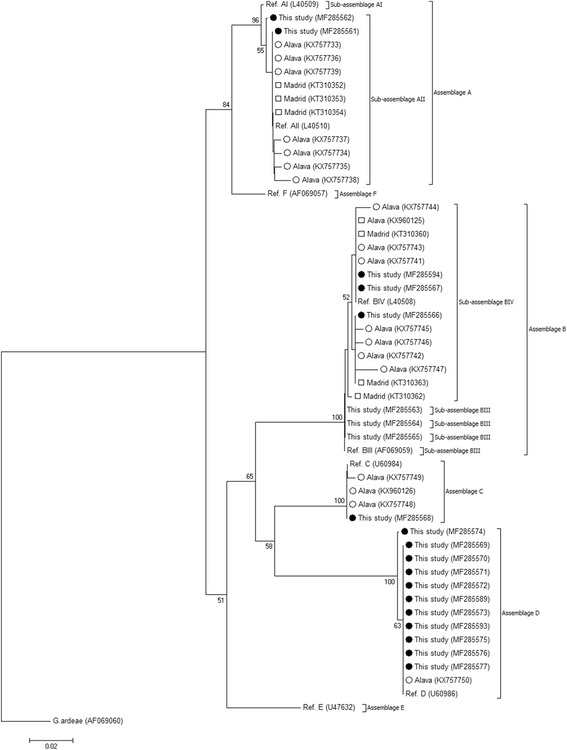
Phylogenetic tree depicting evolutionary relationships among assemblages of *G duodenalis* at the *gdh* locus. The analysis was inferred using the Neighbor-Joining method of the nucleotide sequence covering a 359-bp region (positions 103 to 461 of GenBank accession number L40508) of the gene. Bootstrap values lower than 50% were not displayed. Filled circles represent canine sequences from this study. Open circles and squares indicate canine and human sequences, respectively, reported in other Spanish studies [14, 21, 32]. *Giardia ardeae* was used as outgroup taxa

Sub-genotyping data of the 21 *gdh* sequences with only unequivocal, single-assemblage infections are summarized in Table 2. Two isolates were identified as AII, differing by one to two single-nucleotide polymorphisms (SNPs) with a 405-bp fragment stretching from positions 78–482 of reference sequence L40510. No isolates belonging to sub-assemblages AI or AIII were detected. However, BIII and BIV isolates exhibited a much greater genetic diversity at the nucleotide level. Sequence alignment analyses of BIII isolates with reference sequence AF069059 allowed the identification of a 402 to 412-bp stretch, equivalent to positions 44/54–455 of AF069059. All three BIII isolates differed by seven to 13 SNPs with AF069059, including a high proportion of heterozygous positions (double peaks) detected during chromatogram inspection. Similarly, the two canine isolates assigned to BIV differed by two to four SNPs with a 388/402-bp fragment of reference sequence L40508. Interestingly, one of them (MF285566) showed 100% homology with the second most common BIV genotype (KT310363) detected in clinical patients in Spain [33]. Discordant genotype results BIII/BIV (very likely representing mixed intra-assemblage infections) were detected in four additional isolates (Table 4). The only C isolate genotyped at the *gdh* gene was identical to a 413-bp fragment (positions 78–490) of reference sequence U60984. Finally, the 13 isolates assigned to the assemblage D of *G. duodenalis* were distributed in nine distinct, including a novel (MF285575), genotypes differing between none and four SNPs in a 402-bp fragment stretching from positions 80–481 of reference sequence U60986. The phylogenetic analysis revealed that our A-D sequences clustered together in well-supported clades with the corresponding assemblage and sub-assemblage reference sequences from NCBI, as they also did with sequences of human and canine origin previously documented in other Spanish studies [14, 21, 33] and included here for comparative purposes (Fig. 2).

Sub-genotyping data of the 10 isolates with only single-assemblage infections at the *bg* marker are shown in Table 3. Multiple sequence alignment analyses revealed the presence of four distinct genotypes within AII that varied from one to four SNPs when compared over a ~ 490-bp fragment ranging from positions 97/106 to 587/590 of reference sequence AY072723. One of them (MF285580) corresponded to an AII genetic variant not previously reported. A single AIII isolate was identified, differing in three heterozygous positions from a 488-bp stretch between positions 103–590 of reference sequence AY072724. Importantly, the most prevalent B genotype found in Spanish symptomatic patients (KT310386) [33] was also detected in one of the canine isolates investigated at the *bg* locus in the present study. A novel C genotype (MF285584) revealing two polymorphic sites at positions 37 (G to A) and 451 (C to T) of reference sequence AY545646 was also found, whereas the three isolates genotyped as assemblage D differed from none to four SNPs in a 486-bp stretch (positions 105–590) of reference sequence AY545647.

## Discussion

Few epidemiological surveys have been aimed at investigating the occurrence of *G. duodenalis* in Spanish dog populations. Microscopy-based surveys have estimated the prevalence of *G. duodenalis* at 6–38% in the northeast [18, 19], at 1–10% in the south [20, 22], and at 7–16% in the central areas [23, 24] of the country. Based on the same methodology, *G. duodenalis* cysts have also been found in 5–18% of faecal droppings and soil samples collected in public parks of the latter region [25]. Overall, these figures were well in the range of those reported usually in other European countries [34, 35]. Comparatively higher prevalences of 29–33% have been found in northern Spain when qPCR was the detection method of choice [14, 21], much closer to the 36.5% value identified in the present survey using another high-sensitivity method such as DFAT. Taking together, these data confirm that microscopy examination severely underestimate the actual prevalence of *G. duodenalis* [7]. Of interest, a relatively high proportion (~ 20%) of the canine samples that tested positive by DFAT could not be confirmed by qPCR. This was somehow unexpected, as a previous survey conducted by our lab with stool samples of human origin clearly demonstrated that qPCR had a superior diagnostic sensitivity over DFAT [32]. A potential explanation for this finding is that qPCR failure may be associated, at least partially, to the presence of inhibitory substances in the canine faecal specimens tested.

Our results indicate that *G. duodenalis* infection primarily presented as a sub-clinical, asymptomatic (as suggested by the absence of diarrhoeal episodes) disease in the surveyed canine communities. Although exact age could not be established, the vast majority of the sampled dogs were young adults or mature animals, suggesting that acquired immunity may play a role in the control and/or severity of the infection [8]. Indeed, no recurrence of *Giardia* was reported from dogs older than 1 year which attended at a veterinary hospital in USA during an 11-year period [36]. Indirect support for this phenomenon was provided by i) typically low *G. duodenalis* cyst counts obtained during DFAT examination, and ii) relatively high Ct values obtained during qPCR. Taken together, these findings indicate that low-to-moderate *G. duodenalis* burden was the norm in most of the infected dogs detected in the present study. Importantly, shedding of limited cyst numbers would negatively impact the diagnostic sensitivity of the PCR-based methods used for genotyping and sub-genotyping purposes, a fact further aggravated when considering that both *gdh* and *bg* markers are single-copy genes. Very similar findings have been previously documented in other canine studies in Spain [14, 21].

Marked differences in *G. duodenalis* prevalences were observed among the dog communities investigated here. Sheltered and breeding dogs had a significantly higher prevalence compared to hunting dogs, but not compared to other dog categories. Kennel dogs have been demonstrated to be at higher risk of infection due to continuous exposure to *G. duodenalis* cysts in kennels with high animal density [6, 8, 37]. Of interest was also the high (35.7%) prevalence of *G. duodenalis* observed in pet dogs, a potentially serious public health concern if infected animals are in close contact with children or immunocompromised individuals. Because none of the *G. duodenalis* isolates obtained from pet dogs in the present survey could be genotyped at the assemblage level, more research should be conducted to elucidate the actual role of domestic dogs as natural reservoirs of human giardiosis. Finally, hunting dogs exhibited the lowest (20.6%) *G. duodenalis* infection rate, a figure in the lower range of those (20–30%) reported in similar studies conducted in Spain [19] and Italy [37].

As anticipated, our genotyping analyses confirmed that canine-specific assemblages C and D, particularly the latter, were the most prevalent genetic variants of *G. duodenalis* circulating in dogs in the province of Castellón. Assemblages C/D are known to account for ~ 70% of the cases of canine giardiosis documented in European countries [10, 38, 39], including Croatia [40], Germany [41], Greece [42], and Spain [14, 19]. However, this genotype frequency pattern may vary according to the geographical region and/or the dog population considered. For instance, potentially zoonotic assemblages A/B have been found in 69–89% of the canine isolates genotyped in a number of Spanish [21, 24], German [43], and North American [44] molecular investigations. Remarkably, one in five of our canine infections involved different combinations of *G. duodenalis* assemblages. The fact that the vast majority of these mixed intra-assemblage infections were found in kennelled animals was well in agreement with the former observation that these dogs underwent high infection pressures associated with crowded living conditions (see above). In this regard, mixed intra-assemblage infections have been documented in 2–27% of the canine isolates genotyped in Europe [10] and up to 39% of those from developing countries [13]. In Spain, no mixed *G. duodenalis* infections were demonstrated in two independent molecular surveys based on PCR and sequencing analyses targeting sheltered and pet dogs in the north of the country [14, 21], although an unprecedented mixed infection rate of 48% was allegedly reported in an earlier study based on PCR-RFLP [24].

Sub-genotyping analyses at both the *gdh* and *bg* loci also revealed exciting molecular data. For instance, AII sequences confirmed the high genetic diversity previously reported in canine isolates of this particular sub-assemblage in other Spanish regions [21], with virtually all analysed sequences exhibiting a different pattern of SNP frequency including well-defined point-mutations and heterozygous (double peaks) sites. Interestingly, this phenomenon does not seem to occur in Spanish human isolates, where most (75–100%) of the AII sequences sub-genotyped to date at those very same markers were identical among them [32, 45]. The finding that AII isolates of canine origin present considerably higher levels of heterogeneous nucleotides at both *gdh* and *bg* genes than those of human origin has two important consequences. Firstly, it may supports the existence of genetic exchange, challenging the still widely accepted (but gradually changing) notion that *Giardia* is an organism strictly asexual [46]. In this regard, allelic sequence heterozygosity [47], intragenic recombination [48], and nuclear fusion within cysts [49] have been proposed as potential driving mechanisms of recombination processes in *Giardia*. Additionally, recombination events would be enhanced in epidemiological scenarios characterized by high prevalence rates and elevated infection pressures as those locally described in the present study. Indeed, intra-assemblage recombination has been already demonstrated in AII and B isolates in a highly endemic area in Peru [13, 50]. Secondly, it provides direct molecular evidence disfavouring the role of dogs as natural reservoirs of human giardiosis. Worthy of note was also the identification in a single canine isolate of AIII, a sub-assemblage essentially found in cattle and wild ruminants [10, 11].

Less surprising was the demonstration of high levels of genetic diversity within the canine isolates assigned to sub-assemblages BIII and BIV, in line with previously reported genotyping data in both canine [14, 21] and human [33, 45] populations in Spain and other countries [10, 11]. Notably, two independent canine B isolates were confirmed identical at the *gdh* (MF285566) or the *bg* (MF285583) genes to those predominantly found circulating in Spanish symptomatic patients [33, 45]. This is the only molecular evidence found in the present study backing up dogs as a potential source of human infections in Castellón, although we cannot rule out the possibility of anthroponotic transmission. More moderate, but still relevant, levels of genetic diversity at the nucleotide level were observed within canine-specific assemblages C/D, as clearly demonstrated by the identification of 12 known and one novel assemblage D genotypes.

## Conclusions

Prevalence data presented here are consistent with an epidemiological scenario in which *G. duodenalis* is common in dogs, typically presenting as a light, asymptomatic infection. Highly endemic foci of disease were detected in breeding kennels and dog shelters where infection pressures were high. Although a significant proportion of the infected dogs harboured potentially zoonotic assemblages and sub-assemblages of *G. duodenalis*, most of these genotypes seemed to be primarily transmitted within canine cycles and posed, therefore, a limited risk to humans. However, the actual extent of this statement must be corroborated in future molecular epidemiological surveys including human and dog puppy isolates from this Spanish geographical area.

## Additional files

Additional file 1: Table S1. Oligonucleotides used for the molecular identification and characterization of *Giardia duodenalis* in this study. (DOCX 13 kb)

Additional file 2: Figure S2. Histogram of cycle threshold (Ct) values obtained by real-time PCR for the detection of *Giardia duodenalis* in DNA isolates from canine faecal samples. (DOCX 31 kb)

## Abbreviations

*bg*: ß-giardin
bp: base pair
CI: Confidence interval
Ct: Cycle threshold
DFAT: Direct fluorescent antibody test
DNA: Deoxyribonucleic acid
dNTP: Deoxynucleotide triphosphate
*gdh*: Glutamate dehydrogenase
NCBI: National Center for Biotechnology Information
PCR: Polymerase chain reaction
qPCR: Real-time polymerase chain reaction
RFLP: Restriction fragment length polymorphism
RNA: Ribonucleic acid
SD: Standard deviation
SNP: Single-nucleotide polymorphism
*SSU* rRNA: Small subunit ribosomal RNA
UK: United Kingdom
USA: United Satates of America
