# Supplementary material for: Occurrence and molecular epidemiology of Giardia duodenalis infection in dog populations in eastern Spain
Source: BMC Vet Res. 2018 Jan 22;14:26. doi: 10.1186/s12917-018-1353-z (PMC5778629; doi:10.1186/s12917-018-1353-z)
Supplement: Supplementary file 1 — Oligonucleotides used for the molecular identification and characterization of Giardia duodenalis in this study. (DOCX 13 kb) [file 12917_2018_1353_MOESM1_ESM.docx]

**Supplemental content**

**Supplemental content 1**

Oligonucleotides used for the molecular identification and characterization of *Giardia duodenalis* in this study.

| Target organism | Locus | Oligonucleotide | Sequence (5´–3´) | Reference |
| --- | --- | --- | --- | --- |
| *Giardia duodenalis* | *ssu* rRNA | Probe | FAM–CCCGCGGCGGTCCCTGCTAG–BHQ1 | Verweij et al. (2003) |
|  |  | Gd-80F | GACGGCTCAGGACAACGGTT | Verweij et al. (2003) |
|  |  | Gd-127R | TTGCCAGCGGTGTCCG | Verweij et al. (2003) |
|  | *gdh* | GDHeF | TCAACGTYAAYCGYGGYTTCCGT | Read et al. (2004) |
|  |  | GDHiF | CAGTACACCTCYGCTCTCGG | Read et al. (2004) |
|  |  | GDHiR | GTTRTCCTTGCACATCTCC | Read et al. (2004) |
|  | *bg* | G7 | AAGCCCGACGACCTCACCCGCAGTGC | Lalle et al. (2005) |
|  |  | G759-R | GAGGCCGCCCTGGATCTTCGAGACGAC | Lalle et al. (2005) |
|  |  | G99 | GAACGAACGAGATCGAGGTCCG | Lalle et al. (2005) |
|  |  | G609_R | CTCGACGAGCTTCGTGTT | Lalle et al. (2005) |
