# Supplementary material for: Occurrence and molecular epidemiology of Giardia duodenalis infection in dog populations in eastern Spain
Source: BMC Vet Res. 2018 Jan 22;14:26. doi: 10.1186/s12917-018-1353-z (PMC5778629; doi:10.1186/s12917-018-1353-z)

**Additional file 2: Figure S2**. Histogram of cycle threshold (Ct) values obtained by real-time PCR for the detection of *Giardia duodenalis* in DNA isolates from canine faecal samples.


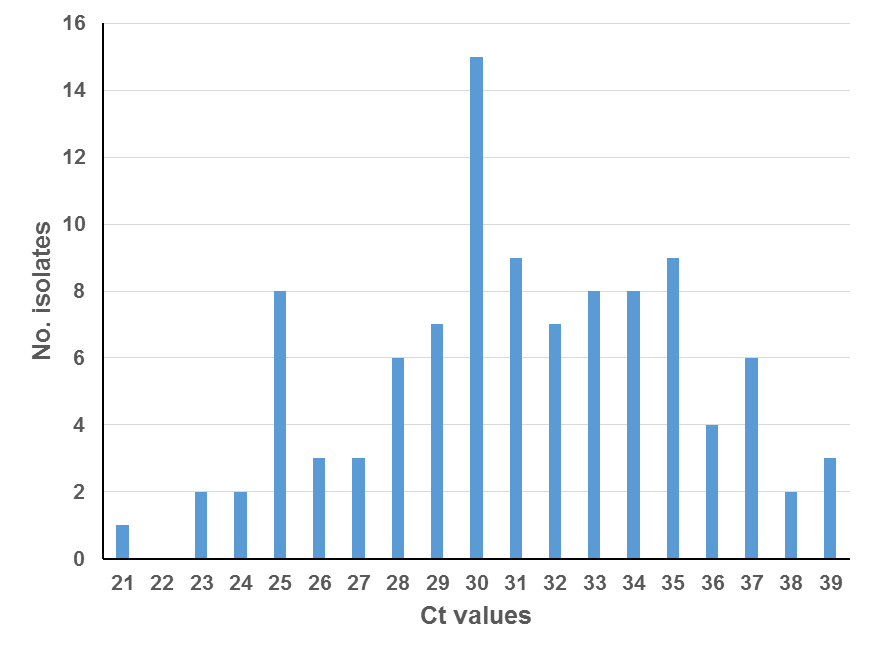

Supplement: Supplementary file 2 — Histogram of cycle threshold (Ct) values obtained by real-time PCR for the detection of Giardia duodenalis in DNA isolates from canine faecal samples. (DOCX 31 kb) [file 12917_2018_1353_MOESM2_ESM.docx]
